# Venous retrograde approach for endovascular angioplasty in chronic total pulmonary vein occlusion -a case report

**DOI:** 10.1186/s12872-024-03984-y

**Published:** 2024-06-22

**Authors:** Bo Li, Hai Zhu, Mengfei Jia, Jinrui Song, Tanba Carl, Gizem Koybasi, Guanming Qi, Hongling Su, Yunshan Cao

**Affiliations:** 1 Department of Cardiology, Pulmonary Vascular Disease Center (PVDC), Gansu Provincial Hospital, 204 Donggangxi Road, Lanzhou, 730000 P. R. China; 2 The First Clinical Medical College of Gansu, University of Chinese Medicine (Gansu Provincial Hospital), Lanzhou, 730000 P. R. China; 3 Department of Cardiology, Shengli Oilfield Central Hospital, 31 Jinan Road, Dongying, 257000 Shandong China; 4 Department of Internal Medicine, Medstar Health, Baltimore, MD USA; 5 Department of Pulmonary Medicine, Yedikule Chest Diseases and Chest Surgery Training and Research Hospital, Istanbul, Turkey; 6 Division of Pulmonary, Critical Care and Sleep, Tufts Medical Center, Boston, MA 02111 USA; 7 Heart, Lung and Vessels Center, Sichuan Provincial People’s Hospital, University of Electronic Science and Technology of China, Chengdu, 610072 Sichuan China

**Keywords:** Venous antegrade approach, Endovascular angioplasty, Pulmonary vein occlusion, Pulmonary vein stenosis, Case report

## Abstract

**Introduction:**

Fibrosing mediastinitis (FM) is a rare disease characterized by excessive proliferation of fibrous tissue in the mediastinum and can cause bronchial stenosis, superior vena cava obstruction, pulmonary artery and vein stenosis, etc.

**Case presentation:**

An aging patient with intermittent chest tightness and shortness of breath was diagnosed with FM associated pulmonary hypertension (FM-PH) by echocardiography and enhanced CT of the chest, and CT pulmonary artery (PA)/ pulmonary vein (PV) imaging revealed PA and PV stenosis. Selective angiography revealed complete occlusion of the right upper PV, and we performed endovascular intervention of the total occluded PV. After failure of the antegrade approach, the angiogram revealed well-developed collaterals of the occluded RSPV-V2b, so we chose to proceed via the retrograde approach. We successfully opened the occluded right upper PV and implanted a stent.

**Conclusions:**

This report may provide new management ideas for the interventional treatment of PV occlusion.

**Supplementary Information:**

The online version contains supplementary material available at 10.1186/s12872-024-03984-y.

## Introduction

Fibrosing mediastinitis (FM), also known as sclerosing mediastinitis or mediastinal fibrosis, is rare but fatal. Progressive exudation and proliferation of fibrinous inflammation in the mediastinum replace normal mediastinal fat, potentially encapsulating and compressing the mediastinal and hilar structures [1, 2]. The median age of FM patients in the United States is 42 years [3], but the mean age is 69.5 years according to a small sample of studies in China [4]. The current general view is that FM is a severe fibrotic change caused by an immune response of the mediastinum to various antigens (e.g., Histoplasma capsulatum, or Mycobacterium tuberculosis) [2]. The immune response leads to mediastinal granulomatous lymphadenitis. The host organism triggers an antigen-antibody response that can lead to mediastinal lymphadenitis [5]. The inflammatory lymph nodes may breakdown, resulting in possible spillage of antigens into the mediastinum. This causes mediastinal allergic reactions and inflammatory changes, and long-term repeated chronic inflammatory stimulation leads to the proliferation of abnormal fibrotic tissue. The large amount of proliferating fibrous tissue may compress the mediastinal structures and cause corresponding pathological changes and clinical manifestations. This process may lead to compression of mediastinal structures and obstructive symptoms. The diagnosis of FM requires the exclusion of other possible diseases of the mediastinum. Patients with FM present with varying degrees of pulmonary vascular narrowing or occlusion and involvement of the accompanying airways. The most common symptoms include cough, chest tightness, shortness of breath, bilateral lower extremity edema, pleural effusion, and clinical features of superior vena cava syndrome when the superior vena cava is compressed. Stenosis and even occlusion of the pulmonary artery (PA) and pulmonary vein (PV) are complications caused by FM [1, 6, 7]. There are relatively few treatment options for FM. Interventional therapy is currently a preferred treatment option. However, it is difficult to treat total PV occlusion, and there is a potentially high risk of procedural-related complications. Here, we report a patient with FM-induced PV occlusion who underwent successful endovascular angioplasty using a venous retrograde approach.

### Case description

**Fig. 1:**
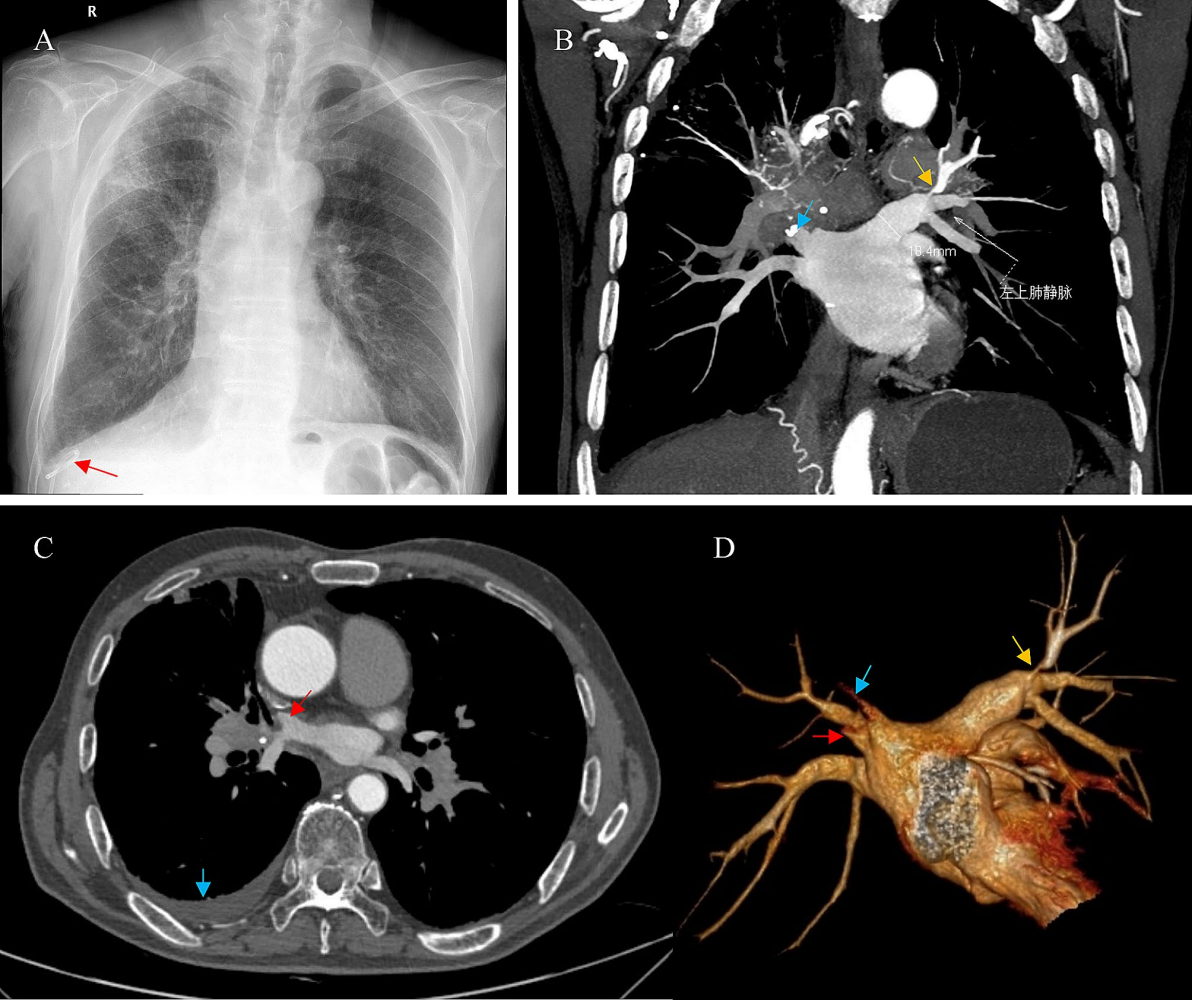
**A**, Chest X-ray image showing the drainage tube in the right lung (red arrow). **B**, Enhanced chest CT: The coronal view shows LSPV stenosis (yellow arrow) and RSPV occlusion (blue arrow). **C**, Enhanced chest CT: Axial view showing RSPV occlusion (red arrow) and pleural effusion (blue arrow). **D**, Virtual reconstructed CT image showing LSPV stenosis (yellow arrow) and RSPV occlusion (blue arrow, V1; red arrow, V2)

A 75-year-old male patient presented with intermittent chest tightness and shortness of breath for more than 1 year. He had a history of paroxysmal atrial fibrillation/flutter, hypertension and tuberculosis. He had no history of smoking or coronary heart disease. Physical examination revealed that the breath sounds decreased in the basal field of the right lung, and the apex was displaced laterally. Laboratory tests revealed an albumin concentration of 35.73 g/l, no abnormalities in liver or renal function, no abnormalities in tumor markers, and an NT-proBNP concentration of 197.80 pg/ml. The pleural effusion was considered leakage. Moreover, no tumor cells were found by pathological examination. Therefore, pleural effusion due to right heart failure or liver, kidney or tuberculosis was excluded. Subsequently, chest X-ray revealed a widened mediastinum. Echocardiography revealed a high probability of pulmonary hypertension (PH), right heart enlargement (right atrial area, 20.1 cm^2^; right ventricular area, 23.2 cm^2^), and moderate tricuspid regurgitation (TRV was 3.7 m/s). The left atrial and ventricular sizes were not abnormal, with normal ranges of systolic and diastolic function and a left ventricular ejection fraction of 63%. The right heart catheter (RHC) suggested elevated mean pulmonary artery pressure (mPAP, 35 mmHg), pulmonary artery wedge pressure (PAWP, 11 mmHg) and pulmonary vessel resistance (PVR, 3.6 Wood units). Enhanced chest computed tomography (CT) with contrast showed multiple enlarged lymph nodes and soft tissue densities around both pulmonary hilum in the mediastinum, multiple stenoses and occlusions of bilateral PAs and PVs (stenosis of the left superior PV (LSPV) and occlusion of the right superior PV (RSPV)), and a large amount of pleural effusion on the right side (Fig. 1). Contrast-enhanced CT showed no significant coronary artery stenosis, and the patient had no symptoms of angina. Accordingly, the patient’s pleural effusion was a result of obstruction of venous return due to PV stenosis/occlusion caused by FM, and leakage of fluid was produced by increased hydrostatic pressure in the pleural capillaries. FM-induced PA/PV stenosis and PH were diagnosed.

**Fig. 2:**
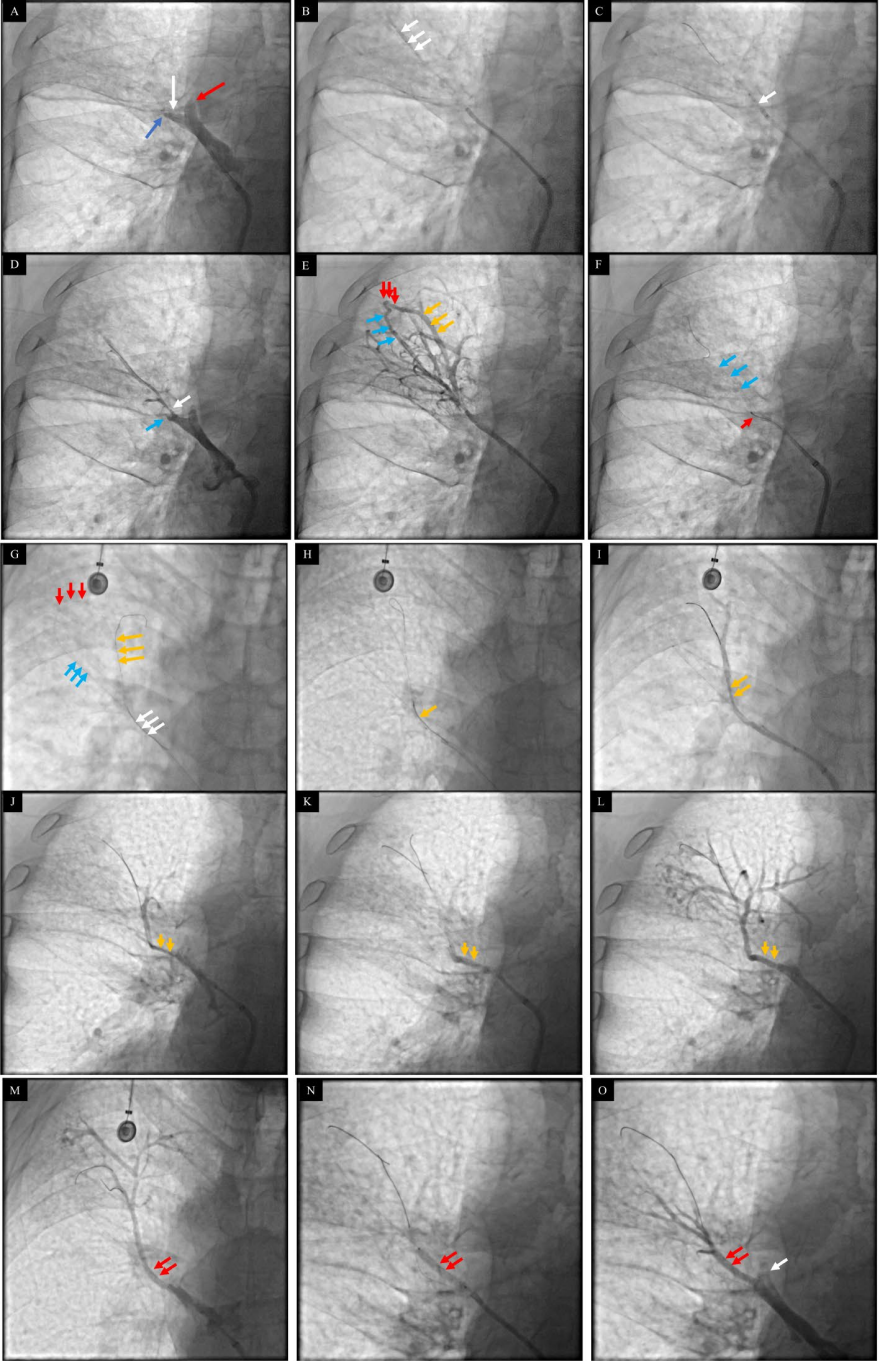
**A**, Selective angiography showing total occlusion of RSPV-V1 (red arrow), total occlusion of RSPV-V2a (white arrow), and RSPV-V2b (blue arrow). **B**, A guidewire with a microcatheter (white arrows) was passed through the occluded segment of the RSPV-V2a and reached its distal end. Angiography via a microcatheter showing the distal V2a (white arrows). **C**, Ballooning (white arrow) of RSPV-V2a. **D**, Selected angiography showing severe stenosis at the proximal segment of RSPV-V2a (white arrow) and occluded RSPV-V2b (blue arrows). **E**, A well-developed collateral vessel (red arrows) connected the distal occluded RSPV-V2b (yellow arrows) to the distal RSPV-V2a (blue arrows). **F**, Failed attempt to pass through the occluded segment of RSPV-V2b (red arrow, guidewire). Blue arrows, the guidewire in V2a. **G**, The guidewire was advanced to the distal area of V2b through the collateral artery (red arrows) and passed through the occluded segment back to the guiding catheter (white arrows). The venous antegrade wire approach was used again, and a Pilot 50 guidewire (yellow arrows) was successfully advanced through the occluded segment to the distal V2a guided by a retrograde wire. **H**, Ballooning (yellow arrows) for V2b. **I**, Selective angiography showed severe stenosis at the proximal V2b (yellow arrows). **J**, Another projection view showing proximal V2b stenosis (yellow arrows). **K**, Stenting for V2b (yellow arrows). **L**, Selective angiography again after stent implantation showing a well-expanded stent (yellow arrows). **M**, Another projection view showing the well-expanded stent (red arrows). **N**, Stenting (red arrows) for V2a. **O**, Selected angiography showing a well-expanded stent in V2a. White arrow, strut of the occluded RSPV-V1

After consulting with multiple disciplinary teams, including cardiac and thoracic surgeons and pulmonologists, endovascular treatment for pulmonary vein stenosis/occlusion was performed. After successfully puncturing the interatrial septum, an 8.5 F vascular sheath (St. Jude Medical, Inc., MN, USA) was advanced to the left atrium, and a JR4.0 guiding catheter (Johnson, NJ, USA) was advanced to the RSPV. Selective angiography revealed total occlusion of the first and second tributaries of the RSPV (RSPV-V1, 2) (Fig. 2A; Supplementary Video A) with pulmonary vein flow grading (PvFG) grade 0. First, the venous antegrade guidewire approach (defined as the guidewire advancing from the proximal to the distal pulmonary vein) failed to pass through the RSPV-V1 occlusion segment. Subsequently, a Pilot 50 guidewire (Abbott, Chicago, USA) with a microcatheter (Finecross^MG^, Terumo Medical, Tokyo, JPN) was passed through the occluded segment of RSPV-V2a and reached its distal end. Serial angioplasty with 1 × 10 mm (Stazuna, Terumo Medical), 1.5 × 15 mm (Springter, Boston Scientific), 2 × 20 mm (Springter, Boston Scientific) and 3 × 30 mm balloons (Sterling™, Boston Scientific) was performed after the true lumen was confirmed by microcatheter angiography (Fig. 2B and C; Supplementary Video A). Angiography revealed severe stenosis in the proximal segment of the RSPV-V2a, the strut of the RSPV-V2b, well-developed collaterals to the occluded RSPV-V2b and a clear-visible distal area of the occluded RSPV-V2b (Fig. 2D and E; Supplementary Video A). After attempting and failing to pass through the occluded segment of RSPV-V2b via the venous antegrade approach (Fig. 2F; Supplementary Video A), we advanced the guidewire to the distal area of V2b through the collateral vessel and passed it through the occluded segment back to the guiding catheter (venous retrograde guidewire approach, defined as the advancement of the guidewire from the distal to the proximal PV) (Fig. 2G; Supplementary Video A). Then, we tried the venous antegrade wire approach again and successfully advanced a Pilot 50 guidewire through the occluded segment to the distal end guided by a retrograde wire (Fig. 2G; Supplementary Video A). After serial ballooning, angiography revealed severe stenosis of the proximal segment of V2b, and a 3-mm diameter stent (3 mm×20 mm, Express™ Vascular SD, Boston Scientific) was implanted (Fig. 2H and M, Supplementary Video A). Additionally, stent implantation was performed in the proximal segment of V2a (Fig. 2N and O; Supplementary Video A). Selective angiography again demonstrated well-expanded stents and no gradient pressures across stents by catheter (Fig. 2L, M and O; Supplementary Video A). No procedure-related complications occurred, and repeat chest CT 12h after stenting showed no lung injury. The symptoms of shortness of breath slightly improved, and the amount of pleural effusion drainage decreased after stenting.

The patient had scheduled follow-ups via phone due to the COVID-19 pandemic, and his symptoms improved compared to those at discharge. The amount of pleural effusion drainage further decreased at the three-month follow-up after stenting. The symptoms improved significantly at the six-month follow-up after the intervention, and the pleural effusion resolved. However, the patient died due to COVID-19-related pneumonia eight months after stenting.

## Discussion

In this case report, we described a patient with PH-FM with intractable pleural effusion. Subsequently, PV stenosis/occlusion was confirmed by chest CT and CT pulmonary venography (CTPV). The severity of PV stenosis is evaluated mainly by imaging, including echocardiography, CTPV/magnetic resonance angiography (MRA), and selective angiography. In recent years, CTPV, selective small pulmonary arteriography and direct imaging of the pulmonary veins have been more frequently applied for comprehensive evaluation [8–10]. In conclusion, the FM dyad (PH signs and pulmonary atelectasis), FM triad (FM dyad plus refractory pleural effusion or upper lobe pulmonary congestion/interstitial pulmonary edema), and FM tetralogy (FM dyad plus pleural effusion and upper lobe interstitial pulmonary edema) have been used as important indications for FM-PVS [11]. There are no specific drugs available for treating PVS, and it is mostly treated symptomatically. Surgical procedures are mostly used for pediatric PVS localized at or near ostia with high restenosis. In FM-PVS, the main focus is mediastinal mass resection, superior vena cava reconstruction, lobectomy, and airway reconstruction. However, operation-related mortality and relapse rates are high [12]. Compared to surgical treatment, endoluminal interventions are less invasive and have better short-term efficacy [13, 14]. Some studies have summarized the indications for PVS intervention based on patients’ clinical manifestations and imaging characteristics, such as single PV proximal stenosis, with a degree > 70% and related symptoms, asymptomatic, with stenosis of the proximal end of both ipsilateral 2 PVs and with a degree > 70%, and severe stenosis of multibranched and multisegmented PVs, which can be treated during the same period or in staged phases one at a time [15–17]. Patients with single-branch PVS, with a degree between 50% and 70%, and no symptoms can be followed up with imaging every 3 to 6 months. In general, the indications for PV intervention were strict due to high rates of intra-stent restenosis. These include the presence of symptoms associated with PVS, such as intractable hemoptysis, intractable pleural effusion, and PH. Stenosis with a single PV is usually based on the gradient between the two ends of the stenosis, with a gradient greater than or equal to 5 mmHg as an indication for intervention.

FM is a rare benign proliferative disease of the fibrous tissue in the mediastinum, but it can be fatal in severe cases. The most common etiologies of FM are Histoplasma capsulatum and Mycobacterium tuberculosis infections, as well as sarcoidosis [18] and autoimmune diseases [19]. There are no common criteria for the diagnosis of FM. Biopsy is the gold standard for the diagnosis of FM [20]. However, due to the invasive nature of biopsies, imaging examinations, such as CTPV and magnetic resonance angiography (MRA), predominate for the diagnosis of FM [21–23]. The imaging features are progressive proliferation of fibrous tissue in the mediastinum and replacement of normal mediastinal adipose tissue, which appears as a focal or diffuse lesion [1, 24]. The proliferating fibrous tissue encapsulates, infiltrates and compresses mediastinal structures, leading to stenosis of large mediastinal vessels and bronchi, such as pulmonary vessels and the superior vena cava, and may also involve other structures in the mediastinum, such as the esophagus [14, 24]. The treatment of FM is a great concern. Currently available drugs for the treatment of FM have been reported but have limited value or insufficient evidence of action. The successful treatment of FM with tamoxifen and steroids has been reported [25]. In a case series of 3 patients with FM, the progression of FM was inhibited by treatment with rituximab in combination with steroid hormones, and the patients were in remission. This result may be because the metabolic activity of the fibrosing mediastinitis lesions was inhibited and its size was reduced due to depletion of B lymphocytes [26]. In addition, a small sample of studies claimed that steroid hormones may play a role in FM for various reasons [27]. However, there is a lack of studies with large samples to support this, and further clinical studies are needed to confirm this.

Tuberculosis-associated FM is more likely to extend bilaterally into the hilar mediastinum. FM can lead to longer compression of the pulmonary vessels, severe narrowing of the lumen, and even occlusion. The bilateral upper PVs are involved earlier and more severely as a result of their proximity to the hilum [28]. PV stenosis may present with segmental pulmonary edema in the corresponding lung tissues or even one-sided or bilateral leaky pleural effusions [11]. PV stenosis/occlusion caused by FM can lead to PH and right heart failure [3]. PV stenosis/occlusion interventional therapy transcatheter PV angioplasty, including balloon angioplasty and stent placement, are currently the main methods of treatment for this fatal disease. The efficacy of the treatment is good, and the stenosis can be immediately resolved to increase the amount of reflux of the PVs and to alleviate the symptoms [14, 29]. Catheter-based therapy has emerged as a promising modality for the treatment of this fatal disease because of the limited efficacy of drug therapy and the high risk of surgery [14]. However, opening PV occlusion is more difficult and could have a greater complication rate because of the pathological features of PV. The retrograde approach has been widely used for coronary artery total occlusion and has been successfully attempted for PA total occlusion [30–32]. The venous retrograde approach could be performed in similar cases where the venous antegrade approach is challenging and well-developed collaterals can be visualized by angiography. Although we were able to open the totally occluded PV via a retrograde approach without any complications and with positive outcomes during the six-month follow-up, the safety of the procedure should be further investigated.

This patient was not able to be followed up in clinic due to the ongoing COVID-19 pandemic, and CTPV was not be performed. Therefore, we were unable to determine whether there was any intrastent restenosis during the follow-up.

In conclusion, the venous retrograde approach for total PV occlusion might be practical but needs to be further studied for its efficacy and safety.

### Electronic supplementary material

Below is the link to the electronic supplementary material.

Supplementary Material 1

Supplementary Material 2

## Data Availability

All the authors confirm that all the data that support the findings of this report are available upon request from the corresponding author.

## Abbreviations

FM: Fibrosing Mediastinitis
PH: Pulmonary Hypertension
CT: Computed Tomography
PA: Pulmonary Artery
PV: Pulmonary Vein
LSPV: Left Superior Pulmonary Vein
RSPV: Right Superior Pulmonary Vein
RHC: Right Heart Catheter
mPAP: mean Pulmonary Artery Pressure
PAWP: Pulmonary Artery Wedge Pressure
LSPV-V1: The first tributary of the LSPV
RSPV-V1, 2: The first and second tributaries of the RSPV
PvFG: Pulmonary Vein Flow Grading
CTPV: Ct Pulmonary Venography
MRA: Magnetic Resonance Angiography
