# Supplementary material for: Venous retrograde approach for endovascular angioplasty in chronic total pulmonary vein occlusion -a case report
Source: BMC Cardiovasc Disord. 2024 Jun 22;24:315. doi: 10.1186/s12872-024-03984-y (PMC11193303; doi:10.1186/s12872-024-03984-y)
Supplement: Supplementary file 1 — Supplementary Material 1 [file 12872_2024_3984_MOESM1_ESM.docx]

**Supplementary materials**

**Supplementary Video legends**

**Supplementary Video A:** **RSPV angiography.**

Serial PV angiography showed the following: (1) the occluded RSPV-V1, RSPV-V2a and RSPV-V2b, (2) the proximal RSPV-V2a stenosis after ballooning and the strut of RSPV-V2b as well as the Well-developed collaterals, (3) retrograde guidewire from RSPV-V2a to V2b, (4) antegrade guidewire to distal RSPV-V2b guided by retrograde guidewire, (5) proximal RSPV-V2b stenosis and well-expanded stents.
